# Structural and Biochemical Characterization of an Atypical α-Carbonic Anhydrase from the Tardigrade *Ramazzottius varieornatus*

**DOI:** 10.3390/molecules31030538

**Published:** 2026-02-03

**Authors:** Byung Hoon Jo

**Affiliations:** 1 Division of Applied Life Science (BK21 Four), Gyeongsang National University, Jinju 52828, Republic of Korea; jobh@gnu.ac.kr; 2 Anti-Aging Bio Cell Factory Regional Leading Research Center (ABC-RLRC), Gyeongsang National University, Jinju 52828, Republic of Korea; 3 Division of Life Science and Research Institute of Life Science, Gyeongsang National University, Jinju 52828, Republic of Korea

**Keywords:** tardigrade, *Ramazzottius varieornatus*, carbonic anhydrase, intrinsically disordered region, surface-exposed cysteine

## Abstract

The tardigrade *Ramazzottius varieornatus* exhibits extraordinary resilience to extreme environmental stresses, yet the functional diversity of its proteome remains largely unexplored. In this study, the structural and biochemical characterization of RvCA5, an atypical α-carbonic anhydrase (CA) identified in *R. varieornatus*, is presented. Expression analysis in *E. coli* revealed the spontaneous formation of a truncated RvCA5 species, which was confirmed to be unrelated to signal peptide cleavage. RvCA5 exhibited distinct structural features, including extended intrinsically disordered regions (IDRs) at both termini. Unlike canonical α-CAs, RvCA5 exhibited negligible CO_2_ hydration activity, which was partially enhanced by the removal of the N-terminal IDR, suggesting that this region acts as a dynamic entropic barrier hindering substrate diffusion. RvCA5 possesses multiple surface-exposed reactive cysteine residues, resembling the redox-sensing human CA 3. Notably, consistent with a predicted nuclear localization signal, in silico modeling predicted that RvCA5 can bind DNA via a positively charged patch near the C-terminal IDR. The DNA-binding capability of RvCA5 was experimentally demonstrated by electrophoretic mobility shift assays. Collectively, these findings suggest that RvCA5 potentially functions as a redox-responsive transcriptional regulator.

## 1. Introduction

The tardigrade, also known as the water bear, is a microscopic animal renowned for its extraordinary ability to withstand extreme physical and chemical stresses, including extreme temperatures, high pressures, radiation, and dehydration. *Ramazzottius varieornatus* is one of the tardigrade species whose genome has been sequenced [1]. Despite available genomic information, research on *R. varieornatus* proteins has been primarily limited to tardigrade-specific intrinsically disordered proteins (TDPs). Dsup (Damage Suppressor) is the most extensively studied TDP; it not only protects the tardigrade’s DNA against irradiation and subsequent oxidative stress but also confers similar radiotolerance to other organisms upon heterologous expression [1,2,3,4,5]. Other TDPs include heat-soluble proteins known to increase desiccation resistance [6,7]. Although recent studies have investigated a limited number of enzymes, such as metabolite phosphatase [8], DNA polymerase [9], mevalonate kinase [10], and superoxide dismutase [11], further research on a broader range of proteins is required to better understand the extraordinary capabilities of *R. varieornatus*.

Carbonic anhydrase (CA, EC 4.2.1.1) is a zinc-containing metalloenzyme that catalyzes the reversible hydration of carbon dioxide (CO_2_): CO_2_ + H_2_O ↔ HCO_3_^−^ + H^+^ [12]. The enzyme-catalyzed reaction is one of the fastest known in biology, with a catalytic rate constant (*k*_cat_) of up to 4.4 × 10^6^ s^−1^ [13]. CA is categorized into eight classes (α, β, γ, δ, ζ, η, θ, and ι) based on discovery order; these classes have evolved via convergent evolution and share almost no sequence homology. CA plays critical physiological roles, including CO_2_ transport and metabolism, biocalcification, pH homeostasis, and photosynthesis [14]. As an essential enzyme ubiquitous across virtually all living organisms, the study of CA is crucial for a broader biological understanding of the host organism. Moreover, CA has gained significant attention as a green biocatalyst for CO_2_ capture and utilization [15]. For this application, CAs from extremophiles or extremotolerant organisms are highly desirable due to their stability under harsh physical (e.g., high temperature) and chemical (e.g., organic solvents) conditions [16]. In this context, the investigation of CA from the extremotolerant *R. varieornatus* would not only broaden fundamental biological insights into this essential enzyme but also provide promising candidate biocatalysts for industrial applications.

Genomic analysis of *R. varieornatus* predicted the existence of ten α-CA (RvCA) isoforms [1]. Previously, six of these isoforms were recombinantly expressed in *Escherichia coli*; however, only RvCA5 could be obtained in sufficient quantities and successfully purified via His_6_-tag affinity chromatography [17]. Herein, the characterization of RvCA5 is reported using a combination of in silico structural prediction and in vitro biochemical assays. RvCA5 showed atypical characteristics, raising intriguing insight about its physiological function.

## 2. Results and Discussion

### 2.1. Sequence Characteristics of RvCA5

**Figure 1:**
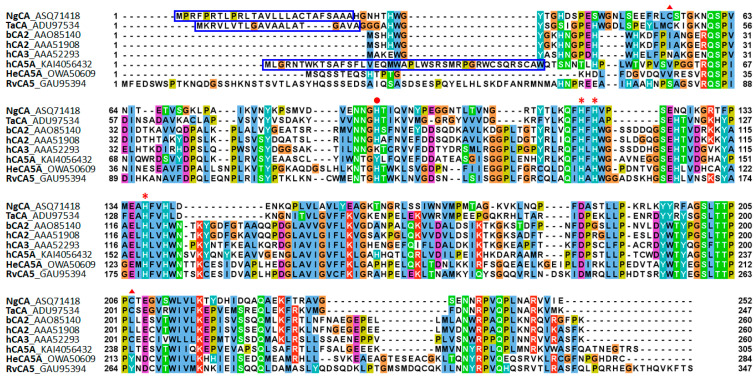
Multiple sequence alignments of selected α-CAs with RvCA5. The predicted signal peptide and the mitochondrial targeting sequence are boxed in blue. The two conserved cysteine residues for the formation of the intramolecular disulfide bond are indicated by a triangle (▲). The three zinc ligand histidine residues (*) and the proton shuttle residue (●) are marked.

Sequence alignment of RvCA5 with selected α-CAs of mammalian and bacterial origins is shown in Figure 1. RvCA5 consists of 347 amino acids and has a theoretical molecular weight of 39.1 kDa (Table 1). The conserved residues, including the three Zn^2+^-coordinating His residues and the proton shuttle His residue, suggest that RvCA is an α-CA [18]. The RvCA5 sequence showed N-terminal and C-terminal extensions not found in the other α-CAs. Any putative signal peptide was not predicted by SignalP6.0.

A BlastP search using the RvCA5 sequence identified two tardigrade homologs: a putative mitochondrial carbonic anhydrase 5A (HeCA5A; GenBank: OWA50609) from *Hypsibius exemplaris* and a carbonic anhydrase 1-like protein (PmCA; GenBank: XP_055357851) from *Paramacrobiotus metropolitanus*, with sequence identities of 62% and 51%, respectively (Supplementary Figure S1a) [19]. When predicted using TargetP 2.0, DeepMito, and Euk-mPLoc, no putative mitochondrial targeting sequence (MTS) was found in RvCA5, HeCA5A, or PmCA, while an MTS was predicted in the sequence of human CA 5A (hCA5A), one of the 16 human α-CA isoforms whose subcellular localization has been experimentally verified [20]. Based on these results, it seems that these tardigrade CAs might not be localized in the mitochondria. Intriguingly, DeepLoc 2.1 identified a nuclear localization signal (NLS) in both the RvCA5 and HeCA5A sequences, suggesting their potential nuclear localization, whereas no NLS was found in the PmCA sequence.

In addition, transcriptomic data indicate that the RvCA5 gene is the most abundantly expressed among the ten RvCA genes (Table 2) [1], suggesting a dominant physiological role in the tardigrade. The mRNA levels of the RvCA5 gene are not significantly altered by dehydration stress and subsequent rehydration process (Table 2), suggesting that RvCA5 is constitutively expressed in the tardigrade.

### 2.2. Expression and Purification of RvCA5 in E. coli

**Figure 2:**
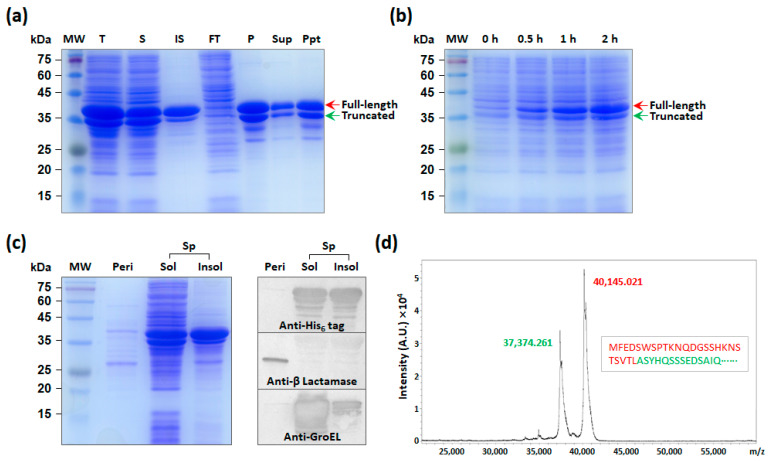
Analysis of recombinant expression, subcellular localization, and truncation of RvCA5 in *E. coli*. (**a**) Expression and purification of RvCA5 analyzed by SDS–PAGE followed by Coomassie Blue staining. Lanes: MW, molecular mass marker; T, total cell lysate; S, soluble fraction; IS, insoluble fraction; FT, flow-through; P, purified enzyme; Sup, supernatant; Ppt, precipitates. (**b**) Time-course analysis of RvCA5 expression. After cell harvest, cells were directly mixed with 4× Laemmli sample buffer and heated for sample preparation. (**c**) Subcellular localization of RvCA5. Lanes: MW, molecular mass marker; Peri, periplasmic fraction; Sp, spherolast fraction; S, soluble fraction; IS, insoluble fraction. (**d**) Mass spectrometric analysis of purified RvCA5.

The RvCA5 gene was chemically synthesized with codon optimization for *E. coli*, cloned into the pET-22b(+) vector, and expressed in *E. coli* BL21(DE3) under the control of the strong T7*lac* promoter induced by the addition of 0.1 mM IPTG at 25 °C, as previously described [17]. The RvCA5 was highly overexpressed, appearing as thick bands on the SDS–PAGE gel with the expected molecular mass (40.1 kDa) (Figure 2a). After cell disruption by sonication, approximately half of the enzyme was found in the soluble fraction from which the RvCA5 was purified using His_6_-tag affinity chromatography. During dialysis against a low-salt phosphate buffer, the precipitation of the RvCA5 occurred, and only a fraction of the enzyme was recovered in the supernatant after low-speed centrifugation (Figure 2a).

### 2.3. Analysis of Truncated RvCA5

Notably, the RvCA5 appeared in two distinct bands on the SDS–PAGE gel (Figure 2a). The small-sized RvCA5 was identified as an N-terminal truncated form of RvCA5, since both proteins, corresponding to the two bands, were purified via the C-terminal His_6_-tag (Figure 2a). The truncated RvCA5 was unlikely to have formed due to internal translation initiation, as no possible translation initiation site with a sufficiently high expression level and the proper reading frame was predicted using the UTR Designer. In addition, the truncation was not due to harsh cell disruption, and the truncated RvCA5 was generated early in the protein expression process and accumulated over time (Figure 2b).

It was hypothesized that the truncated RvCA5 was formed via the rapid but incomplete proteolytic cleavage of the N-terminus shortly after the full-length protein was made. One possibility is that the N-terminal region serves as a signal peptide, directing the entire protein to be exported out of the cytoplasm into the periplasm, where it is then cleaved by a signal peptidase [21]. To confirm this possibility, the periplasmic fraction of the RvCA5-expressing cells was separated from the remaining spheroplast fraction, and then it was determined which fraction contained the RvCA5 proteins. The result showed that the His_6_-tagged RvCA5 was only found in the spheroplast fraction and was absent in the periplasmic fraction (Figure 2c). The Western blot results using the periplasmic marker β-Lactamase and the cytoplasmic marker GroEL confirmed that the fractionation was performed correctly. Since no putative signal peptide was predicted, it was concluded that the truncated RvCA5 was not the result of signal peptide cleavage.

To determine the cleavage site, purified RvCA5 was analyzed by MALDI-TOF mass spectrometry. The analysis yielded two major peaks (Figure 2d): one at approximately *m*/*z* 40,145, corresponding to the full-length RvCA5, and the other at *m*/*z* 37,374, corresponding to the truncated RvCA5. This mass difference indicated that the cleavage occurred specifically between Leucine 25 and Alanine 26. The protease responsible for this specific cleavage, however, remains to be elucidated.

### 2.4. Intrinsically Disordered Regions of RvCA5

**Figure 3:**
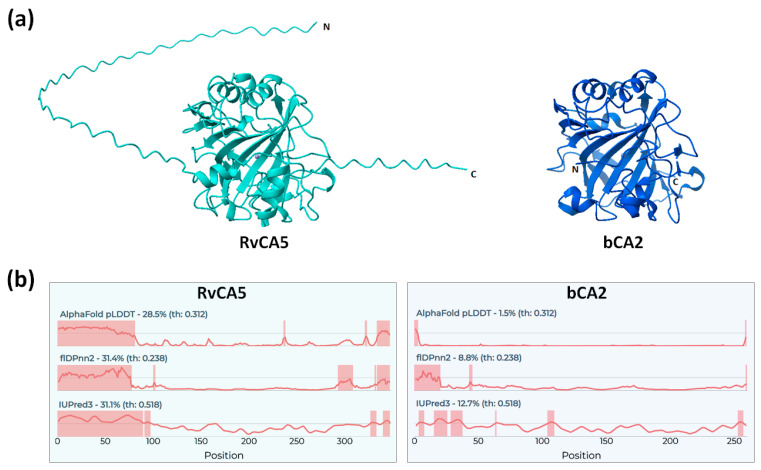
Intrinsically disordered regions (IDRs) of RvCA5. (**a**) Comparison of the RvCA5 structure with the bCA2 structure (PDB ID: 1V9E). (**b**) Sequence-based prediction of IDRs. IDRs are highlighted by shading.

For structural analysis, a predicted structure of the full-length RvCA5 was obtained using AlphaFold (Figure 3a). Consistent with the predicted classification as an α-CA, the structure of RvCA5 was highly similar to those of other α-CAs, such as bCA2, although the core antiparallel β-sheet consisted of 8 strands instead of the typical 10 strands [18]. Notably, the terminal regions exhibited unstructured extensions, which corresponded well to the N- and C-terminal extension sequences (Figure 1). Since the terminal extensions were likely intrinsically disordered regions (IDRs), the intrinsic disorder of RvCA5 was assessed using multiple IDR prediction tools via the CAID prediction portal. As expected, the N- and C-terminal regions were strongly predicted to be disordered, a finding that contrasts sharply with the structure of bCA2 (Figure 3b). The long and unstructured nature of the N-terminal region likely accounts for its susceptibility to proteolytic cleavage. Intriguingly, not only RvCA5 but also all other RvCA isoforms were predicted to possess extended terminal IDRs (Supplementary Figure S2), suggesting that this is a shared characteristic common to all RvCA isoforms.

### 2.5. Activity and Stability of RvCA5

**Figure 4:**
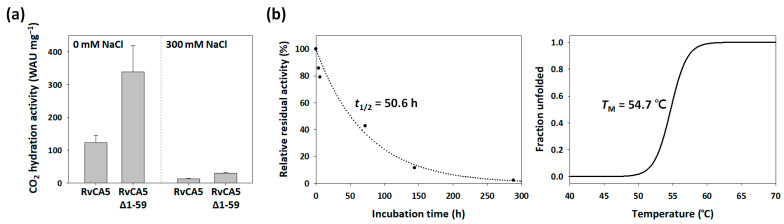
Activity and stability of RvCA5. (**a**) CO_2_ hydration activity of RvCA5 and its derivative RvCA5∆1-59. Enzymes were prepared in 20 mM sodium phosphate buffer (pH 7.5) with or without supplementation of 300 mM NaCl. Error bars represent standard deviations from at least three independent experiments. (**b**) Stability of RvCA5. Kinetic stability (left) was measured based on enzymatic CO_2_ hydration activity after incubation at 40 °C. Data points (closed circles) were fitted to an exponential decay curve to determine the half-life (*t*_1/2_). The thermodynamic stability (right) was estimated by temperature-dependent circular dichroism spectroscopy.

The activity of RvCA5 was determined by the CO_2_ hydration assay. The RvCA5 showed CO_2_ hydration activity of 123.4 ± 22.0 WAU mg^−1^ (Figure 4a), which is only 1% of the activity of bCA2 (12,700 WAU mg^−1^) [14]. When the RvCA5 was prepared in a buffer containing 300 mM NaCl, the activity decreased by an order of magnitude (to 13.0 ± 0.6 WAU mg^−1^) compared to the activity of RvCA5 prepared in a buffer without NaCl supplementation (Figure 4a). This result suggests that RvCA5 is susceptible to salt-induced denaturation. The ICP-OES analysis showed that recombinant RvCA5 contains approximately 0.54 moles of zinc per mole of enzyme. Since Zn^2+^ coordination within the α-CA active site is strictly governed by the precise geometric arrangement of three conserved histidine residues [22], the observed 54% zinc occupancy confirms that the enzyme was properly folded. This indicates that the remarkably low activity is not a consequence of structural instability or a deficiency of the zinc cofactor, but is instead an intrinsic functional characteristic of RvCA5. Given its extremely low CO_2_ hydration activity, RvCA5 is unlikely to function primarily in CO_2_ metabolism, unlike typical CA enzymes.

Moreover, the RvCA5 showed moderate stability with a half-life of 50.6 h at 40 °C and a melting temperature of 54.7 °C (Figure 4b), contradicting the initial anticipation that it would be an extremozyme. Recent studies have demonstrated that tardigrades in their active hydrated state are not inherently thermostable, and their exceptional thermal tolerance is primarily a feature of the desiccated state [23,24]. Thus, individual enzymes in tardigrades are likely susceptible to thermal stress, and the stability results for RvCA5 are consistent with the organism’s actual physiological limits in its active state.

### 2.6. Expanding the Substrate Tunnel Improves the Activity of RvCA5

**Figure 5:**
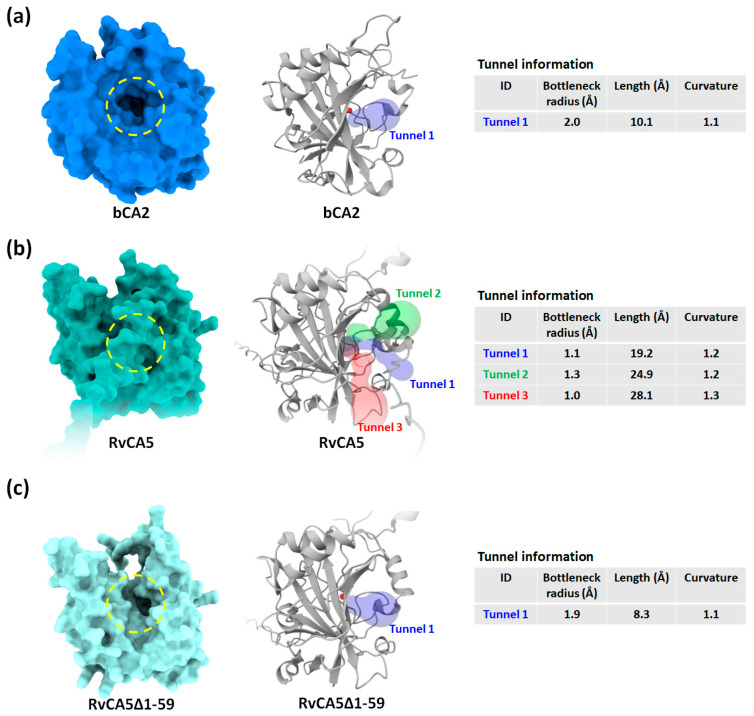
Analysis of substrate tunnel in (**a**) bCA2, (**b**) RvCA5, and (**c**) RvCA5∆1-59, an N-terminal IDR-deleted derivative of RvCA5. The yellow dashed circle represents the cone-shaped cavity.

Next, the substrate tunnel of the enzyme was analyzed using Caver Web 2.0. α-CAs generally possess a deep, cone-shaped tunnel through which diffusion of substrates and products into and out of the buried active site occurs. The typical tunnel architecture of α-CA is also present in bCA2, manifesting as a deep (~10 Å) cavity extending close to the active site (Figure 5a) [25]. In contrast, a large area of the tunnel entrance on RvCA5 was blocked by a part of the N-terminal IDR, thereby reshaping it into three narrow and longer tunnels instead of a single wide one (Figure 5b). While this structural analysis is based on a static snapshot, it is important to consider the highly dynamic nature of IDRs. Through rapid conformational fluctuations, the IDR can occupy a large excluded volume around the tunnel entrance [26], acting as a dynamic entropic barrier that hinders the diffusion of CO_2_ molecules into the active site.

Based on the hypothesis that the extremely low activity of RvCA5 was (at least partially) due to inefficient mass transfer, an N-terminal IDR-deleted version of the enzyme (RvCA5∆1-59) was constructed to test for improved activity. The removal of the blocking region successfully reverted the tunnel architecture to one closely resembling that of bCA2, featuring a much larger bottleneck radius (1.9 Å) and a shorter depth (8.3 Å) when compared to the original RvCA5 (Figure 5c). The CO_2_ hydration activity of RvCA5∆1-59 was more than two-fold higher compared to the original RvCA5, regardless of NaCl supplementation (Figure 4a). Furthermore, the zinc content of RvCA5∆1-59 was determined to be approximately 0.58 moles of zinc per mole of enzyme, a value closely comparable to the 0.54 mol/mol measured for the original RvCA5. These results collectively demonstrate that the significant increase in enzymatic activity upon the removal of the N-terminal IDR is primarily attributable to enhanced mass transfer, rather than any variation in zinc occupancy within the active site.

### 2.7. Unusual Surface Exposure of Cysteines

**Figure 6:**
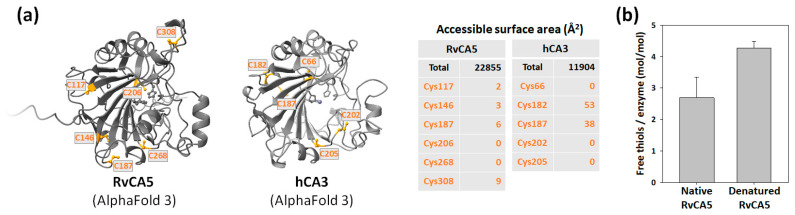
Assessment of surface-exposed cysteines. (**a**) Localization of surface-exposed cysteines on RvCA5 and hCA3. Residues are indicated in orange, and corresponding solvent-accessible surface area values are tabulated. (**b**) Quantitative analysis of free thiols in RvCA5 under native and denatured conditions using Ellman’s assay.

Many α-CAs, particularly of bacterial origin, have a conserved intramolecular disulfide bond (Figure 1) [12]. The other nine RvCAs, except for RvCA5, also possess the two corresponding cysteine residues required to form this conserved disulfide bond (Table 1). However, RvCA5 is distinct from the other RvCAs in that it lacks the two cysteine residues for the conserved disulfide bond and has a total of six cysteine residues, an exceptionally large number found in α-CAs. The six cysteines are spatially separated in the RvCA5 structure, preventing the formation of an intramolecular disulfide bond (Figure 6a).

The cytosolic human CA 3 (hCA3) has a very low catalytic activity (<1% of the fastest isozyme hCA2) [27]. Notably, hCA3 possesses five cysteine residues, among which two reactive cysteines are surface-exposed and susceptible to oxidative modifications such as S-glutathionylation (Figure 6a) [28,29]. hCA3 is believed to function as an antioxidant agent, utilizing its reactive cysteines to protect cells from oxidative damage. In the case of RvCA5, four of the six cysteine residues were predicted to be surface-exposed, albeit the corresponding solvent-accessible surface areas were calculated to be smaller than those of hCA3 (Figure 6a). Quantification of free thiols using Ellman’s assay revealed that native RvCA5 possesses 2.7 free thiols per enzyme, confirming the presence of surface-exposed cysteine residues (Figure 6b). In its denatured state, the number of free thiols increased to 4.3 per enzyme. This value is lower than the theoretical total of 6 cysteines, likely due to partial oxidation of surface cysteines, resulting in the formation of intermolecular disulfide bonds. The observed difference in thiol content between the native and denatured states is presumably attributable to the two buried cysteines, Cys206 and Cys268. The presence of multiple surface-exposed cysteines raises the intriguing possibility that RvCA5 may function as a potential sensor that responds to changes in cellular redox status. Notably, the tardigrade homologs HeCA5A and PmCA also possess multiple surface-exposed cysteines (Supplementary Figure S1a,b). Furthermore, these three tardigrade CAs share two conserved, surface-exposed cysteine residues (Cys187 and Cys308 in RvCA5 numbering), implying a potential functional conservation of redox-sensitive regulation across these tardigrade species.

### 2.8. Interaction of RvCA5 with DNA

**Figure 7:**
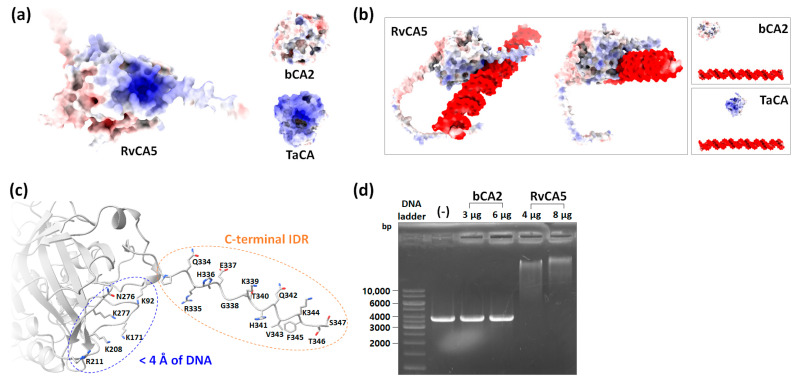
Interaction of RvCA5 and DNA. (**a**) Electrostatic surface potential map of CAs at pH 7, represented on a scale from −10 kT/e (red) to +10 kT/e (blue). (**b**) Prediction of DNA binding using AlphaFold. (**c**) Residues in RvCA5 that are potentially involved in DNA interaction. The six residues within 4 Å of DNA and the 15 residues of the C-terminal IDR are highlighted in blue and orange, respectively. (**d**) Experimental validation of DNA binding by EMSA. (-), negative control with DNA only.

Although RvCA5 is a slightly acidic protein with a pI value of 6.85, the analysis of surface electrostatic potential revealed a positively charged surface patch near the C-terminal IDR (Figure 7a). Since an NLS was identified in RvCA5, it was assumed that the positively charged surface patch might be involved in DNA binding. Accordingly, the binding of RvCA5 to DNA was tested in silico using the AlphaFold server with an arbitrary dsDNA sequence (5′-ATACCTAGGAACATTTAGATACAGGGCCTTAGATGACTCTGATGCTAGCTAGC-3′). Surprisingly, the prediction showed that RvCA5 could bind to DNA via a positively charged surface patch (Figure 7b). Consistent results were observed across multiple random DNA sequences. In contrast, bCA2 lacks such a positively charged patch and was predicted not to bind to DNA (Figure 7a,b). TaCA, a bacterial periplasmic α-CA from *Thermovibrio ammonificans* that possesses positively charged surface patches, was also tested to exclude the possibility that the predicted binding of RvCA5 to the DNA was due to nonspecific electrostatic interactions. The result showed that TaCA did not interact with the DNA (Figure 7a,b), suggesting that the predicted DNA binding was specific to RvCA5. In RvCA5, the majority (5 out of 6) of the residues found within 4 Å of DNA were positively charged Lys and Arg residues that are crucial for electrostatic attraction to negatively charged DNA (Figure 7c). Notably, the C-terminal IDR extending near the DNA has residues that are potentially important for DNA binding: positively charged residues (R335, H336, K339, H341, K344) for electrostatic interactions with DNA backbone, and polar and nonpolar residues (Q334, E337, T340, Q342, V343, F345, T346, S347) for sequence recognition and binding specificity. Regarding the tardigrade homologs, PmCA was predicted to bind DNA, whereas HeCA5A was not (Supplementary Figure S1c,d). Given that no NLS was identified in the PmCA sequence, its predicted DNA-binding activity warrants further validation in future studies.

To experimentally validate RvCA5’s predicted DNA-binding activity, an electrophoretic mobility shift assay (EMSA) was performed. Incubation with purified RvCA5 induced a concentration-dependent migration shift in the linearized plasmid DNA (3741 bp). In contrast, bCA2 showed no interaction with the DNA substrate under the same conditions (Figure 7d). These results demonstrate a physical interaction between RvCA5 and DNA, although the specific physiological target sequences remain to be identified.

### 2.9. Putative Cellular Function of RvCA5

The structural and functional profile of RvCA5 identified in this study—namely, its low catalytic activity, multiple surface-exposed cysteines, terminal IDRs, and DNA-binding capability—suggests a cellular role distinct from typical enzymatic functions. RvCA5 may have evolved as a moonlighting protein by repurposing the ancestral CA scaffold for alternative functions [30]. The combination of low catalytic activity and surface cysteines closely parallels the characteristics of hCA3, a known cellular redox sensor [28,29]. Based on these structural features, a hypothesis is proposed that RvCA5 may function as a potential redox-responsive transcriptional regulator. In this model, RvCA5 is putatively translocated into the nucleus upon redox activation, where it may bind DNA via its positively charged surface patch. The C-terminal IDR might contribute to DNA-binding specificity through a disorder-to-order transition, while the N-terminal IDR could potentially serve as a flexible scaffold for cytoplasmic redox partners or recruit transcription factors to modulate gene expression. The abundant, constitutive expression of RvCA5 likely ensures an immediate functional response to environmental stresses. Given that terminal IDRs are common across the RvCA isoforms, these IDRs may also help stabilize proteins under extreme desiccation, as do other TDPs. Intriguingly, although the homologs HeCA5A and PmCA exhibit predictive profiles for NLS and DNA-binding ability that differ from those of RvCA5, they all share multiple surface-exposed cysteines. This conservation suggests that while the redox-sensing scaffold may be maintained across the tardigrade species, downstream mechanisms may have diverged or specialized across different tardigrade lineages. Although this putative mechanism remains to be experimentally confirmed, it provides a plausible framework for future in vivo validation of the physiological roles of RvCA5 in *R. varieornatus* and other tardigrade homologs.

## 3. Materials and Methods

### 3.1. Vector Construction

The *Escherichia coli* TOP10 strain was used for the construction and amplification of plasmid vectors, and the *E. coli* BL21(DE3) strain was used for the overexpression of recombinant proteins. *E. coli* cells were cultivated in a shaking incubator (Jeiotech, Daejeon, Republic of Korea) at 37 °C and 180 rpm in Luria–Bertani (LB) medium, added with 50 μg/mL ampicillin whenever necessary. For recombinant expression of RvCA5 (GenBank accession number: GAU95394), the previously constructed pET-RvCA5 vector was used, in which the RvCA5 gene is inserted into the pET-22b(+) vector via *Nde*I and *Xho*I restriction sites [17]. For the expression of the truncated version of RvCA5 (∆1-59), the corresponding DNA fragment was obtained from the pET-RvCA5 vector by PCR using forward primer 5′-CATATGCGTATGAACATGGCGC-3′ and reverse primer 5′-CTCGAGGCTGGTAAATTTC-3′ (*Nde*I and *Xho*I restriction sites underlined), and was subcloned into the pET-22b(+) vector, resulting in the pET-trncRvCA5 vector. For the expression of bovine CA 2 (bCA2), the bCA gene was chemically synthesized (Genscript, Piscataway, NJ, USA) and subcloned into pET-22b(+) using *Nde*I and *Xho*I restriction sites, yielding pET-bCA. All the genes had the His_6_-tag sequence at their 3′ terminus.

### 3.2. Expression and Purification of Recombinant Enzymes

The constructed vectors were each introduced into *E. coli* BL21(DE3), and the recombinant strains were cultured at 37 °C in LB medium supplemented with ampicillin. When the optical density at 600 nm (OD_600_) of the culture reached 0.6 as measured via a spectrophotometer (UV-1800; Shimadzu, Kyoto, Japan), 0.1 mM isopropyl-β-D-thiogalactopyranoside (IPTG; Duchefa Biochemie, Haarlem, The Netherlands) was added to the medium to induce recombinant protein expression driven by the T7*lac* promoter. Additionally, 0.1 mM ZnSO_4_ (Junsei, Kyoto, Japan) was added to supplement Zn^2+^, the cofactor required for CA activity. Following 12 h of post-induction cultivation at 25 °C, the cells were harvested by centrifugation at 4 °C and 4000× *g* for 10 min. The cells were resuspended in lysis buffer (50 mM sodium phosphate, 300 mM NaCl, and 10 mM imidazole; pH 8.0) and subsequently disrupted using an ultrasonic homogenizer (Sonics and Materials, Newtown, CT, USA) while kept on ice. Then, the lysate was centrifuged at 4 °C and 10,000× *g* for 10 min, and the supernatant and the remaining debris were designated the soluble and insoluble fractions, respectively. For the affinity purification of recombinant enzymes, the soluble fraction was mixed with Ni^2+^-nitrilotriacetic acid agarose beads (Qiagen, Germantown, MD, USA), and the His_6_-tagged recombinant enzyme was purified according to the manufacturer’s instructions by sequentially applying wash buffer (50 mM sodium phosphate, 300 mM NaCl, and 30 mM imidazole; pH 8.0) and elution buffer (50 mM sodium phosphate, 300 mM NaCl, and 250 mM imidazole; pH 8.0). Finally, the eluates were thoroughly dialyzed with a dilution factor of at least 10^−11^ against enzyme buffer (20 mM sodium phosphate; pH 7.5) at 4 °C using a Spectra/POR dialysis membrane with a 6–8 kDa MWCO (Spectrum Labs, Compton, CA, USA). If necessary, the enzyme buffer was supplemented with 300 mM NaCl.

### 3.3. SDS–PAGE and Western Blot

Protein samples were mixed with 4× Laemmli sample buffer (Bio-Rad, Hercules, CA, USA) and heated for 10 min at 100 °C. They were separated by sodium dodecyl sulfate-polyacrylamide gel electrophoresis (SDS–PAGE) on a 15% PAGE gel and visualized by Coomassie blue R-250 (Bio-Rad, Hercules, CA, USA) staining. For periplasmic fractionation, the harvested cells were partially lysed and separated into the periplasmic fraction (Peri) and the spheroplast fraction (Sp) using the osmotic shock method as previously described [31]. For Western blot analysis, proteins separated by SDS–PAGE were transferred to a nitrocellulose membrane (Whatman, Piscataway, NJ, USA), and primary and secondary antibodies were applied sequentially. Mouse monoclonal anti-His_6_ antibody (ABM, Vancouver, BC, Canada), rabbit polyclonal anti-β-lactamase antibody (Merck, Rahway, NJ, USA), and rabbit polyclonal anti-GroEL antibody (Enzo Life Sciences, Farmingdale, NY, USA) were used as the primary antibodies. Rabbit polyclonal anti-mouse IgG and goat anti-rabbit IgG antibodies (Bethyl Laboratories, Montgomery, TX, USA), both conjugated to alkaline phosphatase, were used as the secondary antibodies. Alkaline phosphatase activity was chromogenically detected using nitroblue tetrazolium (NBT) and 5-bromo-4-chloro-3-indolyl phosphate (BCIP) (Surmodics, Eden Prairie, MN, USA).

### 3.4. Mass Spectrometry

The purified RvCA5 enzyme solution was thoroughly dialyzed against deionized water before mass spectrometric analysis. The molecular mass of the purified RvCA5 was analyzed by matrix-assisted laser desorption/ionization time-of-flight (MALDI-TOF) mass spectrometry (Autoflex Speed; Bruker Daltonics, Billerica, MA, USA), operated in linear mode.

### 3.5. Protein Quantification

For protein quantification, the purified enzymes were denatured in denaturing buffer (6 M guanidine hydrochloride (GuHCl)/20 mM sodium phosphate buffer; pH 7.5), and the absorbance of the denatured protein was measured at 280 nm in a quartz crystal cuvette (Hellma Analytics, Müllheim, Germany). The protein concentration was determined from the measured absorbance using the molar extinction coefficient of RvCA5 at 280 nm, calculated by ProtParam (http://web.expasy.org/protparam/, accessed on 22 June 2025) [32].

### 3.6. CA Activity Assay

CA activity was measured using a colorimetric Wilbur-Anderson CO_2_ hydration assay performed at 0 °C within a UV-1800 spectrophotometer equipped with a temperature-controlled cell holder, as previously described [14]. Briefly, 5–50 μL of the enzyme sample at 20 μM was added to a disposable cuvette (Sarstedt, Lower Saxony, Germany) containing 600 μL of assay buffer (20 mM Tris, 100 μM phenol red; pH 8.3). The CO_2_ hydration reaction was initiated by adding 400 μL of CO_2_-saturated deionized water, which was prepared in ice-cold water, to the cuvette. The absorbance change was monitored at 570 nm, and the time (*t*) required for the pH drop from 7.5 to 6.5 was obtained. The time (*t*_0_) for the uncatalyzed reaction was also measured by adding the same volume of the corresponding blank buffer. The Wilbur-Anderson unit (WAU) was calculated as (*t*_0_−*t*)/*t*.

### 3.7. Zinc Content Analysis

The zinc content of purified enzyme was quantified by inductively coupled plasma optical emission spectroscopy (ICP-OES) on an Optima 8300 DV instrument (PerkinElmer, Waltham, MA, USA).

### 3.8. Thermostability Test

For the kinetic stability test, the samples were heated at 40 °C in a water bath (Jeiotech, Daejeon, Republic of Korea) for up to 12 days. They were then stored at 4 °C until enzyme activity was measured using the CO_2_ hydration assay. The relative residual activity was calculated based on the activity of the unheated sample as follows:

$$Relative\;residual\;activity\left(\%\right)=\left(\frac{WAU\;of\;heated\;sample}{WAU\;of\;unheated\;sample}\right)\times 100$$

For the thermodynamic stability test, a circular dichroism (CD) spectrum was obtained using a CD spectropolarimeter (Jasco, Tokyo, Japan). The temperature of the RvCA5 solution inside a quartz cuvette was increased from 25 °C to 70 °C at a rate of 2 °C/min by heating. The change in the ellipticity was recorded at 220 nm, and the obtained data were fitted to a Boltzmann sigmoidal equation using SigmaPlot 10.0 (Systat Software, San Jose, CA, USA).

### 3.9. Free Thiol Quantification

Free thiol content was measured according to Ellman’s assay [33]. First, 250 µL of RvCA5 solution was added to a reaction solution containing 700 µL of denaturing buffer. Subsequently, 50 µL of 2 mM 5,5’-dithiobis(2-nitrobenzoic acid) (DTNB; Sigma-Aldrich, St. Louis, MO, USA), dissolved in 20 mM sodium phosphate buffer (pH 7.5), was added to the reaction solution. After the reaction was allowed to proceed for 5 min, the mixture was transferred to a quartz cuvette (Hellma Analytics), and the absorbance was measured at 412 nm. For blank correction, 20 mM sodium phosphate buffer (pH 7.5) was added instead of the enzyme sample. The measured absorbance was used to calculate the free thiol concentration using the extinction coefficient (13,600 M^−1^ cm^−1^) of 2-nitro-5-thiobenzoic acid. For measurement under native conditions, the same volume of 20 mM sodium phosphate buffer (pH 7.5) was used instead of the denaturing buffer.

### 3.10. Electrophoretic Mobility Shift Assay (EMSA)

A pGEM-T easy vector (Promega, Madison, WI, USA) harboring the superfolder GFP gene flanked by a HindIII site was linearized using the HindIII restriction enzyme (ThermoFisher Scientific, Waltham, MA, USA) to generate a 3741 bp dsDNA. The linearized DNA was purified via agarose gel electrophoresis using the FavorPrep^TM^ Gel/PCR purification kit (FAVORGEN, Ping Tung, Taiwan) and eluted in deionized water. For the binding assay, 10 µL of the DNA solution (approximately 200 ng) was mixed with an equal volume of the enzyme solution (10 µM or 20 µM) prepared in 20 mM sodium phosphate buffer (pH 7.5), and the mixture was incubated for 1 h at room temperature. The samples were then mixed with ExcelDye^TM^ 6× DNA loading dye (SMOBIO, Hsinchu City, Taiwan) and resolved on a 1% agarose gel with TAE buffer (40 mM Tris, 1.14 mL glacial acetic acid, 1 mM EDTA) and subsequently stained with StaySafe^TM^ Nucleic Acid Stain (RBC Bioscience, New Taipei City, Taiwan). DNA bands were visualized using the GelDoc Go imaging system (Bio-Rad, Hercules, CA, USA).

### 3.11. In Silico Analyses

Multiple sequence alignment was performed using ClustalX2.0 [34] and visualized using Jalview 2.11.5.1 [35]. Signal peptide prediction was performed using SignalP 6.0 [36]. Mitochondrial targeting sequence was predicted using TargetP 2.0 [37], DeepMito [38], and Euk-mPLoc 2.0 [39]. Nuclear localization signal was identified using DeepLoc 2.1 [40]. The translation initiation region was predicted using the UTR Designer [41]. Protein structure was predicted using the AlphaFold 3 server [42] and visualized with UCSF ChimeraX 1.9 [43]. Intrinsically disordered regions (IDRs) and binding regions inside IDRs were predicted using the CAID prediction portal [44]. Analysis of tunnels in protein structures was performed using the CAVER Web server [45] with default values for CAVER parameters, except for the following parameters: shell depth (20 Å), shell radius (10 Å), and clustering threshold (5 Å). The solvent-accessible surface area was assessed using the XSSP server [46]. Electrostatic potential on protein surface was calculated by Adaptive Poisson–Boltzmann Solver (APBS) [47].

## Figures and Tables

**Table 1:** Protein parameters of RvCAs.

Name (RvY ID, UniProt ID)	Protein Length	Molecular Weight (kDa)	No. Cysteine Residues ^a^		pI
			Conserved	Non-Conserved (Surface-Exposed)	
RvCA1 (RvY_03263, A0A1D1UN98)	323	36.6	2	0 (0)	9.27
RvCA2 (RvY_01739, A0A1D1UL76)	354	39.5	2	0 (0)	5.91
RvCA3 (RvY_01195, A0A1D1UQT7)	414	46.4	2	1 (1)	8.62
RvCA4 (RvY_04176, A0A1D1UXK6)	364	41.4	2	1 (1)	6.25
RvCA5 (RvY_07018, A0A1D1V0M4)	347	39.1	0	6 (4)	6.85
RvCA6 (RvY_06685, A0A1D1UZF6)	347	38.7	2	0 (0)	6.17
RvCA7 (RvY_13508, A0A1D1VTD1)	333	38.3	2	2 (2)	8.30
RvCA8 (RvY_17267, A0A1D1W3U6)	383	42.5	2	4 (4)	7.11
RvCA9 (RvY_09162, A0A1D1V8H4)	325	37.3	2	1 (1)	8.16
RvCA10 (RvY_13109, A0A1D1VUC0)	344	39.0	2	2 (2)	5.90

^a^ Signal peptides were excluded from the calculation.

**Table 2:** Expression profile of RvCA genes obtained by mRNA sequencing [1].

Name (RvY ID, UniProt ID)	Expression (FPKM)		
	Active	Dehydrated	Rehydration (3 h)
RvCA1 (RvY_03263, A0A1D1UN98)	5.7	3.3	3.0
RvCA2 (RvY_01739, A0A1D1UL76)	23.9	17.3	19.8
RvCA3 (RvY_01195, A0A1D1UQT7)	48.0	41.8	43.5
RvCA4 (RvY_04176, A0A1D1UXK6)	2.3	2.8	2.9
RvCA5 (RvY_07018, A0A1D1V0M4)	276.3	256.6	273.3
RvCA6 (RvY_06685, A0A1D1UZF6)	32.4	28.9	32.0
RvCA7 (RvY_13508, A0A1D1VTD1)	14.7	10.4	13.4
RvCA8 (RvY_17267, A0A1D1W3U6)	23.4	23.7	24.7
RvCA9 (RvY_09162, A0A1D1V8H4)	4.5	4.0	5.2
RvCA10 (RvY_13109, A0A1D1VUC0)	19.3	16.9	16.4

## Data Availability

The original contributions presented in this study are included in the article/Supplementary Material. Further inquiries can be directed to the corresponding author.

## Supplementary Materials

The following supporting information can be downloaded at https://www.mdpi.com/article/10.3390/molecules31030538/s1. Figure S1: Comparison of RvCA5 with homologous tardigrade CAs. HeCA5A, carbonic anhydrase from Hypsibius exemplaris; PmCA, carbonic anhydrase from Paramacrobiotus metropolitanus. (a) Multiple sequence alignments. Cysteine residues are boxed in yellow, and the conserved, surface-exposed cysteines (▼) are marked. (b) Assessment of surface-exposed cysteines. Cysteine residues are indicated in orange, and corresponding solvent-accessible surface area values are tabulated. The conserved, surface-exposed cysteines are marked in red. (c) Electrostatic surface potential map of CAs at pH 7, represented on a scale from −10 kT/e (red) to +10 kT/e (blue). (d) Prediction of DNA binding using AlphaFold. Figure S2. Structure of RvCA isoforms predicted by AlphaFold.
