# Supplementary material for: Structural and Biochemical Characterization of an Atypical α-Carbonic Anhydrase from the Tardigrade Ramazzottius varieornatus
Source: Molecules. 2026 Feb 3;31(3):538. doi: 10.3390/molecules31030538 (PMC12899995; doi:10.3390/molecules31030538)
Supplement: Supplementary file 1 [file molecules-31-00538-s001.zip › molecules-4084216-supplementary.pdf]

Supporting Information

Structural and Biochemical Characterization of an Atypical α-Carbonic Anhydrase from the Tardigrade *Ramazzottius varieornatus*

Byung Hoon Jo [1,2,3]

[1] Division of Applied Life Science (BK21 Four), Gyeongsang National University, Jinju 52828, Republic of Korea; jobh@gnu.ac.kr
[2] Anti-Aging Bio Cell Factory Regional Leading Research Center (ABC-RLRC), Gyeongsang National University, Jinju 52828, Republic of Korea
[3] Division of Life Science and Research Institute of Life Science, Gyeongsang National University, Jinju 52828, Republic of Korea

Supplementary Figures

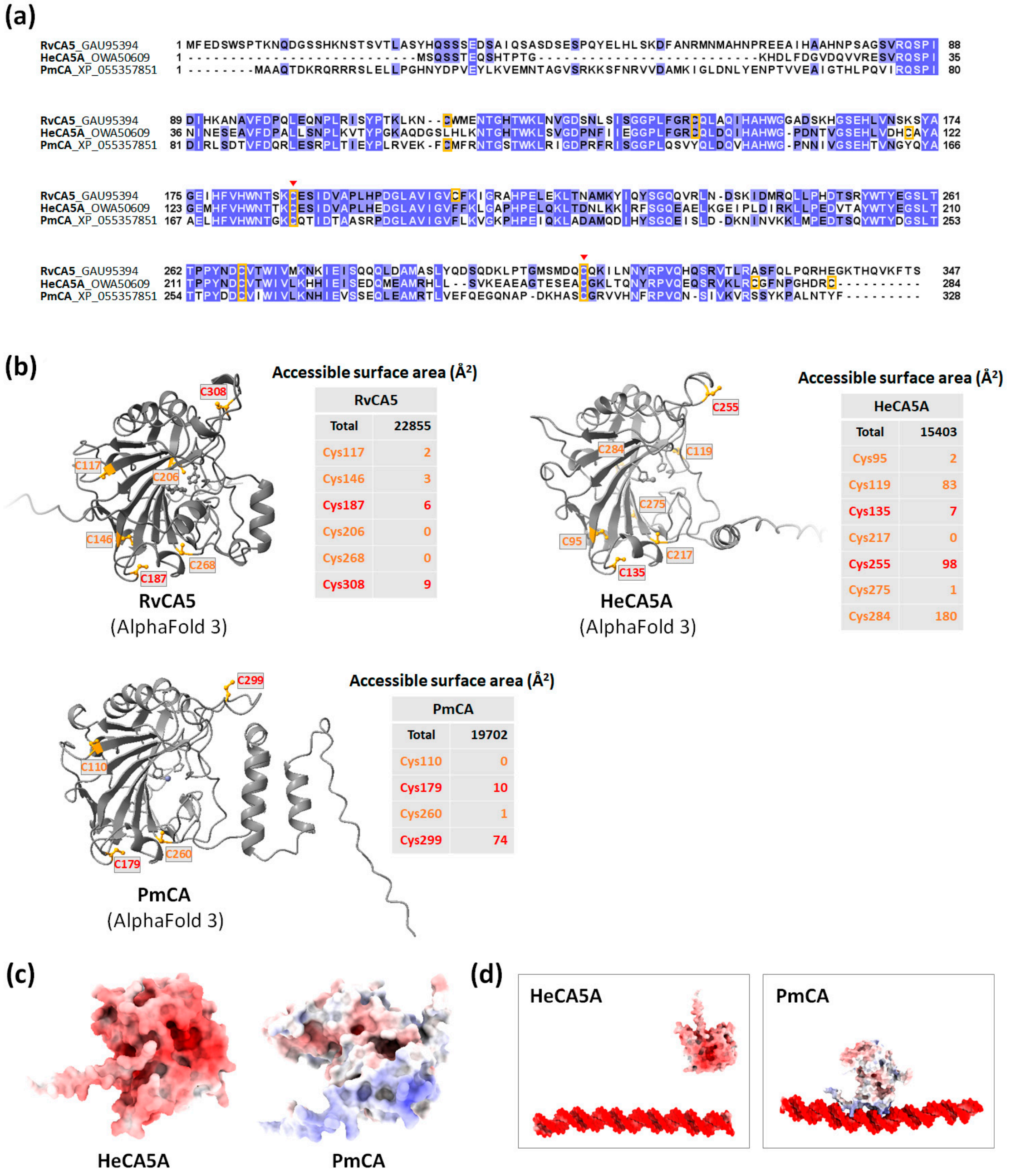

Figure S1. Comparison of RvCA5 with homologous tardigrade CAs. HeCA5A, carbonic anhydrase from *Hypsibius exemplaris*; PmCA, carbonic anhydrase from *Paramacrobiotus metropolitanus*. (**a**) Multiple sequence alignments. Cysteine residues

are boxed in yellow, and the conserved, surface-exposed cysteines (▼) are marked. (**b**) Assessment of surface-exposed cysteines. Cysteine residues are indicated in orange, and corresponding solvent-accessible surface area values are tabulated. The conserved, surface-exposed cysteines are marked in red. (**c**) Electrostatic surface potential map of CAs at pH 7, represented on a scale from -10 kT/e (red) to +10 kT/e (blue). (**d**) Prediction of DNA binding using AlphaFold.

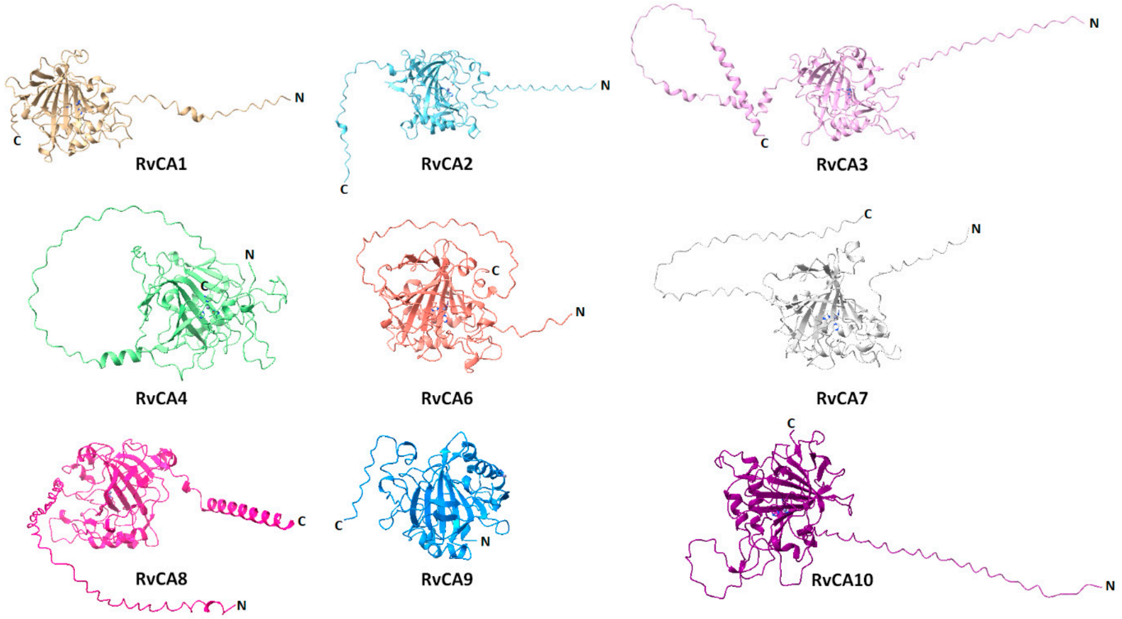

Figure S2. Structure of RvCA isoforms predicted by AlphaFold.
